# Rhabdomyolysis Following Initiation of Posaconazole Use for Antifungal Prophylaxis in a Patient With Relapsed Acute Myeloid Leukemia: A Case Report

**DOI:** 10.1177/2324709617690747

**Published:** 2017-02-01

**Authors:** Mayur D. Mody, Deepak Ravindranathan, Harpaul S. Gill, Vamsi K. Kota

**Affiliations:** 1 Emory University, Atlanta, GA, USA

**Keywords:** posaconazole, rhabdomyolysis, AML, acute myeloid leukemia

## Abstract

Posaconazole is a commonly used medication for antifungal prophylaxis in patients with high-risk acute leukemia, such as acute myeloid leukemia. Despite clinical data that show that posaconazole is superior to other antifungal prophylaxis medications, posaconazole is known to have many side effects and drug-drug interactions. We present a patient who developed rhabdomyolysis after being started on posaconazole for prophylaxis in the setting of relapsed acute myeloid leukemia.

## Introduction

Patients with neutropenia resulting from chemotherapy for acute myeloid leukemia (AML) are oftentimes at high risk for invasive fungal infections.^1^ Systemic review and meta-analysis review data have shown that invasive fungal infections occur in 5% to 40% of patients with hematologic malignancies and are most commonly seen in AML, with invasive aspergillosis and candidiasis being the most common pathogens.^2,3^ Data from a 2016 cohort study of 250 AML patients found 16 cases (6.4%) of probable/proven invasive fungal infections and 44 cases (17.6%) of possible invasive fungal infections.^4^ A recent systematic review and meta-analysis that included 64 randomized trials to evaluate the effect of systemic antifungal prophylaxis in cancer patients after chemotherapy or hematopoietic stem cell transplantation showed significantly decreased all-cause mortality, fungal-related mortality, and documented invasive fungal infections when systemic antifungal prophylaxis was used.^5^

The use of antifungal agents for prophylaxis, specifically azoles, has become standard of care for high-risk acute leukemia patients, including those undergoing AML induction chemotherapy. Azoles work by inhibiting the cytochrome P450-dependent enzyme lanosterol 14-α-demethylase, an enzyme that is necessary for the conversion of lanosterol to ergosterol, a vital component of the cellular membrane of fungi. The disruption of this conversion causes significant damage to the cell membrane by increasing its permeability, ultimately resulting in cell lysis and death.^6^ The considerations and risks of antifungal prophylaxis include drug toxicities, selection for resistant pathogens, adverse drug-drug interactions, and costs.^7^ We present the case of a patient found to have severe rhabdomyolysis following the initiation of posaconazole for antifungal prophylaxis in the setting of relapsed AML being treated with induction chemotherapy.

## Case Description

A 55-year-old male with a past medical history significant for hypertension, gastroesophageal reflux disease, benign prostatic hypertrophy, and relapsed AML diagnosed 1 year prior presented to a community hospital with a 1-day history of fevers and chills with highest temperature recorded at 100.7°F at home. Of note, the patient had been discharged from a National Cancer Institute–designated Cancer Center 10 days prior to this presentation. During this previous 8-day hospitalization, patient received inpatient re-induction chemotherapy with FLAG-IDA after bone marrow biopsy showed relapsed AML inv 16, c-kit negative with 30% blasts. He tolerated the chemotherapy without any major complications. The patient did not require any packed red cell transfusions during this prior admission, but did receive 1 unit of platelets for platelet count of 28 000/µL prior to discharge. He was discharged on acyclovir, levofloxacin, pentamidine (patient has a reported Bactrim allergy), and posaconazole (patient was to start this medication following discharge) for prophylaxis of infection in the setting of neutropenia. The patient endorsed compliance with this medication regimen in the interval between his discharge from the National Cancer Institute–designated Cancer Center to his presentation at the community hospital.

During initial evaluation at the community hospital, he was found to be febrile with temperature of 103.1°F and tachycardic to the 130s. Labs revealed leukopenia with white blood cell count of 100/µL, anemia with hemoglobin of 8.5 g/dL, and thrombocytopenia with platelet count of 10 000/µL. His chemistry panel, including electrolytes and renal function, were all within normal limits. He was given intravenous fluids, started on broad-spectrum antibiotics, and admitted for sepsis in the setting of neutropenic fever and pancytopenia.

The patient was continued on cefepime and vancomycin, as well as his prophylactic antiviral and antifungals. Oncology was consulted and recommended platelet transfusion for thrombocytopenia and filgrastim for leukopenia. Infectious workup, including urinalysis (UA), chest X-ray, influenza testing, and blood cultures were negative.

**Figure 1.**
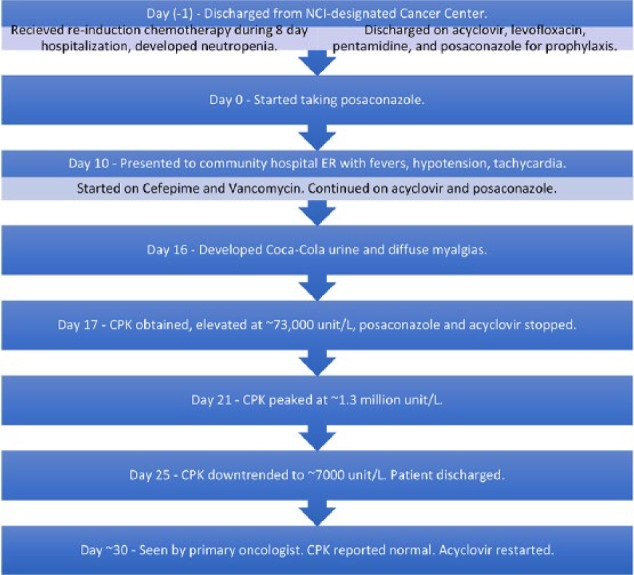
Case presentation: summary of timeline of events.

On approximately day 6 of admission, the patient started noticing that his urine had turned Coca-Cola in color. He also complained of diffuse myalgias which had started the day prior. A UA was performed on day 7 of admission and showed 2+ protein and 3+ blood, neither of which were seen on UA obtained on admission. A serum creatine phosphokinase (CPK) was obtained and found to be significantly elevated at 73 133 unit/L (reference range 35-232 unit/L). Nephrology was consulted and recommended mannitol and bicarbonate in addition to intravenous fluids for treatment of rhabdomyolysis. The patient’s renal function remained normal throughout the admission. However, the patient’s CPK continued to rise, peaking at 1 318 326 unit/L on day 11 of admission, prior to downtrending to 7493 unit/L at the time of discharge (Figure 1). The rhabdomyolysis was attributed to being an adverse effect of the patient’s prophylaxis antimicrobials, specifically posaconazole, which had recently been started. Posaconazole and acyclovir were both discontinued prior to discharge, and patient was restarted on acyclovir only during follow-up with his oncologist less than a week following his discharge.

## Discussion

Posaconazole is 1 of the 5 members of the triazole class licensed for use in the United States, along with fluconazole, itraconazole, voriconazole, and isavuconazole.^6^ Posaconazole was initially only available as an oral suspension. In 2013, the US Food and Drug Administration approved delayed-release tablets for the prophylaxis of invasive *Aspergillus* and *Candida* infections in patients at high risk for these infections, such as patients with hematologic malignancies with prolonged chemotherapy-induced neutropenia.^8^ The introduction of the extended-release tablet form addressed one of the major limitations of the oral suspension form—its variable bioavailability.^9^ The oral suspension form of the drug had already been found superior to fluconazole and itraconazole in 2 randomized controlled trials in AML patients undergoing chemotherapy. In a landmark 2007 study by Cornely et al^1^ of AML/MDS patients undergoing induction chemotherapy, patients who received oral suspension posaconazole (n = 304) were less likely than those receiving oral itraconazole (n = 58) or oral fluconazole (n = 240) to develop invasive fungal infections (2% vs 8%, *P* < .001), and had lower 100-day mortality (14% vs 21%, *P* = .04). A second multicenter randomized control trial of 252 AML patients by Shen et al^10^ in 2013 showed similar results, as the use of posaconazole was associated with a lower rate of probable/proven invasive fungal infections than oral fluconazole (4% vs 9%, *P* = .026).

Despite studies showing its superiority to other azole agents (posaconazole has not yet been compared to voriconazole or echinocandins in AML patients not undergoing hematopoietic cell transplantation), and the introduction of the extended-release tablet form, limitations to posaconazole still remain. Specifically, posaconazole’s interaction with cytochrome P450 enzymes and P-glycoprotein complicates its use in patients requiring multiple concomitant transplant-related medications or newer targeted therapies for leukemias such as isocitrate dehydrogenase inhibitors.^6^ In addition to these drug-drug interactions, common toxicities of posaconazole include gastrointestinal effects (18%), headache (5% to 17%), fever (12%), dry mouth (9%), neutropenia (7%), musculoskeletal pain (7%), and liver toxicity (5%).^11,12^

We present a case of a patient that developed rhabdomyolysis approximately 2 weeks following the initiation of the extended-release tablet form of posaconazole, with a CPK level peaking at 1 318 326 unit/L. While there have been no case reports of posaconazole-induced rhabdomyolysis in the literature, musculoskeletal pain is a common toxicity of the drug. Our patient was not on any drugs that strongly interfere with the 3A4 isoform of the cytochrome P450. Our patient was taking tamsulosin for his benign prostatic hypertrophy, and while posaconazole may increase serum concentrations of this drug due to its inhibitory effects on CYP3A4, the major metabolic pathway for tamsulosin, tamsulosin does not have any known musculoskeletal side effects.^13^ Similarly, carvedilol is partly metabolized by CYP3A4, so drug concentration alterations of carvedilol may also have been possible.^14^ Of note, prior to the patient’s presentation to the emergency room, he was noting low blood pressures at home, and so he was holding his carvedilol. While this could be solely due to sepsis, an interaction between tamsulosin and posaconazole and/or carvedilol and posaconazole may have also been contributing to the hypotension.

Due to improved bioavailability of the extended-release tablet form of posaconazole, routine plasma drug levels are not indicated. The patient was on appropriate prophylactic dosing of the drug, making a toxic serum level less likely. While no known major drug interactions exist between posaconazole and antimicrobial agents such as acyclovir, pentamidine, levofloxacin, vancomycin, and cefepime, it is important to note that the patient was on all of these agents around the time of the development of his rhabdomyolysis.

## Conclusion

AML patients undergoing induction chemotherapy are at high risk for invasive fungal infections. Posaconazole has been shown to have a lower rate of invasive fungal infections compared to fluconazole and itraconazole, at the risk of potentially higher incidence of serious triazole-related adverse events.^6^ The recent introduction of the extended-release tablet form of posaconazole has led to its increased use for prophylaxis purposes in AML patients with chemotherapy-related neutropenia. Given the significant interactions of posaconazole with cytochrome P450 enzymes and P-glycoprotein, the testing of new generation, broad-spectrum triazoles such as isavuconazole for prophylaxis purposes in AML patients is underway.^15^ We present the case of a patient with relapsed AML undergoing re-induction therapy that developed severe rhabdomyolysis shortly after initiation of posaconazole. Our case received a score of 5 on the Naranjo scale, inferring that the patient’s rhabdomyolysis was a probable adverse drug reaction of posaconazole. Although the recommendation for monitoring of plasma posaconazole drug levels does not pertain to patients receiving the tablet form of the drug, as in our patient, cautionary use should still be used given the potential for serious complications such as in our patient, particularly when a patient is on other medications.^16^
